# Probabilistic mortality forecasting with varying age-specific survival improvements

**DOI:** 10.1186/s41118-016-0017-8

**Published:** 2017-01-12

**Authors:** Christina Bohk-Ewald, Roland Rau

**Affiliations:** 1 Max Planck Institute for Demographic Research, Konrad-Zuse-Straße 1, 18057 Rostock, Germany; 2 University of Rostock, Ulmenstrasse 69, 18057 Rostock, Germany

**Keywords:** Mortality forecasts, Rates of mortality improvement, Prediction intervals, Bayesian inference, Coherent mortality forecasts

## Abstract

Many mortality forecasting approaches extrapolate past trends. Their predictions of the future development can be quite precise as long as turning points and/or age-shifts of mortality decline are not present. To account even for such mortality dynamics, we propose a model that combines recently developed ideas in a single framework. It (1) uses rates of mortality improvement to model the aging of mortality decline, and it (2) optionally combines the mortality trends of multiple countries to catch anticipated turning points. We use simulation-based Bayesian inference to estimate and run this model that also provides prediction intervals to quantify forecast uncertainty. Validating mortality forecasts for British and Danish women from 1991 to 2011 suggest that our model can forecast regular and irregular mortality developments and that it can perform at least as well as other widely accepted approaches like, for instance, the Lee-Carter model or the UN Bayesian approach. Moreover, prospective mortality forecasts from 2012 to 2050 suggest gradual increases for British and Danish life expectancy at birth.

## Introduction

Few activities of demographers receive as much attention from society as population forecasts. Many areas of public policy are affected by the increasing number of the elderly in a population as a result of improved chances of survival (Preston and Stokes 2012). Financing old-age pensions and health care and the provision of long-term care are only the tip of the iceberg. However, not only public policy is affected. Private companies also require reliable estimates for future mortality. Pension funds represent the most obvious example (OECD 2011; Soneji and King 2012).

Despite their great societal relevance, the recent track record of demographic forecasts is not praiseworthy. Even though longer time series became available as well as presumably better methodology and more computing power were on-hand, Keilman (2008) concluded for European countries that “Demographic Forecasts Have Not Become More Accurate Over the Past 25 Years.” Projecting only mortality—as one of the three parameters, besides fertility and migration, that determine the size of a population and shape its age-structure—does not yield satisfactory results either (Keilman 2008, p. 146).

The seminal method of Lee and Carter (1992) generates robust mortality forecasts using a parsimonious principal component model that captures age and period effects. Numerous extensions of this model have been developed to increase the predictive performance (see for an overview, e.g., Booth (2006), Booth et al. (2006), Booth and Tickle (2008), Shang et al. (2011, 2012), and Butt and Haberman (2010a). There are also more flexible approaches such as the P-spline approach of Currie et al. (2004), approaches using gaps from best-practice life expectancy at birth (e.g., Torri and Vaupel 2012), and approaches using cause of death data (e.g., Oeppen 2008), as well as approaches that use information from exogenous factors such as tobacco smoking (e.g., Janssen et al. 2013), GDP, and alcohol consumption (e.g., Girosi and King 2008; French and O’Hare 2014). Latest developments in mortality forecasting increasingly include Bayesian inference (see information below), and they account for the distribution of the age at death in order to capture detailed patterns of mortality (Janssen and de Beer 2016; Basellini and Camarda 2016).

Although many of these approaches can yield better forecasts than the original Lee-Carter model, it is still challenging to forecast unsteady mortality developments such as turning points in the long-term trend (Coelho and Nunes 2011) or the aging of mortality decline (Horiuchi and Wilmoth 1995). Nevertheless, some recently developed approaches address this issue using different modeling strategies. For example, they extend the Lee-Carter predictor structure to let the age pattern rotate with time (e.g., Li et al. 2013; Ševčíková et al. 2016), use multiple principal components in the product-ratio functional approach (e.g., Hyndman et al. 2013; Hyndman and Ullah 2007), use mortality trends of multiple populations (e.g., Russolillo et al. 2011; Li and Lee 2005), or use rates of improvement rather than death rates to forecast variable mortality changes (e.g., Haberman and Renshaw 2012; Mitchell et al. 2013).

The objective of this paper is to propose a model that newly combines three of these different modeling strategies in order to forecast mortality for developed countries, particularly in the presence of changing long-term trends and dynamic age-shifts of survival improvement. The modeling strategies include the following:

### (a) Rates of mortality improvement (ROMIs)

As noted above, Mitchell et al. (2013) and Haberman and Renshaw (2012) use ROMIs to forecast variable mortality changes. They take the predictor structure of the Lee-Carter framework and replace the log death rates with their corresponding rates of improvement. Both approaches argue that they can yield better forecasting results than the original Lee-Carter model and some of its variants. We also use ROMIs in the proposed model; we define them as the time-derivative of age-specific death rates. In our opinion, there are two main reasons that justify using ROMIs. Firstly, while the actual level of death rates determines current values of life expectancy at birth, it is the age-specific rate of change that determines the development into the future. It has been demonstrated in the past that the pace of survival improvement is rather independent of the current level of mortality (Vaupel 1997; Kannisto et al. 1994). Secondly, it is now well established that life expectancy at birth is rising for more than 170 years (e.g., Oeppen and Vaupel 2002; White 2002; Tuljapurkar et al. 2000; Vallin and Meslé 2009). Despite the linear pattern in the increase of life expectancy at birth, mortality did not decrease at all ages simultaneously. In many (highly) developed countries, infant and childhood ages contributed most to the increase in life expectancy at birth in the nineteenth and the early twentieth century; nowadays, life expectancy at birth rises primarily because of reductions in mortality among the oldest-old (Christensen et al. 2009). Death rates are falling now at very advanced ages (e.g., above 85 and even above 90), where mortality was often considered to be fixed (Rau et al. 2008; Vaupel 2010), at such a rapid pace that even purely data-driven models were unable to capture the trend (e.g., Currie et al. 2004). We expect that modeling ROMIs instead of death rates can enable the proposed model to capture underlying mortality dynamics (often better than benchmark models); incorporating the aging of mortality decline might help to increase the quality of predictions.

### (b) Combine mortality trends of multiple countries

Recent studies give evidence that it can be advantageous to jointly forecast mortality of multiple populations. For instance, Cairns et al. (2011) forecast co-moving mortality for two populations, whereas Russolillo et al. (2011) and Li and Lee (2005) forecast mortality of a single country using a shared trend among a group of countries. The assumption behind this methodology is convergence of mortality. It can be justified for a group of countries experiencing sufficiently homogeneous mortality such as (highly) developed countries or western industrialized countries. A reason for converging mortality could be, for instance, that populations who are in the same stage of health transition belong to one group. The theory of Health Transition has been proposed by Frenk et al. (1991), and it is used, for example, by Vallin and Meslé (2004) in order to explain converging and diverging mortality trends for multiple countries across time. The proposed model can, in this regard, use the mean mortality trend of reference countries (RCs) to complement the mortality trend of a single country of interest (COI). This is especially useful if a forecaster anticipates that mortality in a COI will increasingly converge to mortality in RCs (that belong to the same group). For instance, the long-term increase of Danish female life expectancy at birth has been interrupted by a period of almost stagnation during the 1980s and early 1990s. A simple extrapolation of this stagnating trend would lead to an underestimation of the progress in mortality that has been observed thereafter. The proposed model could capture this turning point, if it modeled the Danish mortality trend to increasingly converge to the one of Sweden. Sweden also belongs to western countries, but it has experienced lower mortality and greater survival improvements than Denmark at the same time. Supporting this argument, Vallin and Meslé (2004) suggested that Denmark may enter a new phase of convergence to levels and trends of mortality in western industrialized countries (such as Sweden) from the early 1990s onwards. Hence, in the presence of (anticipated) long-term trend changes, we think that it can be worthwhile for a model to (be able to potentially) assume that the trend of a COI will increasingly approach the trend of RCs (that belong to the same group as the COI) during forecast years. The anticipation of long-term trend changes, and the selection of RCs is, incontestably, subjective. How subjective information and expert judgment can be differentiated in (demographic) forecasting is discussed, for example, in general in Armstrong (2001) and more specifically in Billari et al. (2014a). In order to avoid an arbitrary character of choosing RCs, a possible strategy may be to take a larger group of populations that share major mortality risk factors and survival conditions such as western industrialized countries. This strategy would be similar to, for example, the applied world mean in the Bayesian approach of Raftery et al. (2013) or to the mean of leading countries that is used in the mortality forecasts of EUROSTAT, EUROPOP 2010 and 2013 (European Commission 2014).

### (c) Probability statements

We use simulation-based Bayesian inference to estimate and run the proposed model; it automatically captures and quantifies forecast uncertainty with probability statements. Despite the early contribution of Törnqvist in the middle of the twentieth century (Alho and Spencer 2005), generating mortality forecasts with probability statements using (simulation-based) Bayesian inference is still relatively new. For instance, Girosi and King (2008) and King and Soneji (2011) use Bayesian inference to forecast age-specific mortality (by cause of death) as a dependent variable that is influenced by multiple explanatory variables like sex, location, and GDP. Raftery et al. (2013) revise the traditional deterministic approach from the UN to forecast life expectancy at birth. This model has recently been revised by Ševčíková et al. (2016) in order to account for aging of mortality decline. The UN approach combines country-specific and overall country information with a time series approach. Czado et al. (2005), Pedroza (2006), and Kogure and Kurachi (2010) use Bayesian inference to revise the Lee-Carter model in order to address problems with, for instance, erratic data, projection uncertainty, and missing data. Wiśniowski et al. (2015) propose a comprehensive Bayesian population projection model that also uses revised versions of the Lee-Carter model to forecast vital rates and migration. Billari et al. (2012; 2014b) propose a Bayesian model to forecast a population probabilistically, using opinions from experts regarding the future development of vital rates as input data in order to determine, e.g., their (expected) median values, marginal variability, and correlation structure across time.

Using the three modeling strategies, i.e., ROMIs, converging mortality, and Bayesian inference, the proposed model is supposed to tackle two issues (that are particularly important in developed countries): Firstly, dynamic age shifts in mortality decline and, secondly, turning points in long-term trends in forecast years.

The remainder of our paper is organized as follows: First, we formally introduce the mortality forecasting model, i.e., its input, core, and output. Thereafter, we apply the proposed model to forecast mortality for women and men in the UK and Denmark. We select these two countries, because they represent two general forecasting situations in terms of the presence of turning points in the long-term mortality trend. While the increase in life expectancy at birth was rather uniform for British women and men, it included long-term trend changes for Danish women and men. We use these different mortality trends to assess the performance of the proposed model in validating forecasts, and to compare its predictive ability with that of the model of Lee and Carter (1992), some of its variants (Renshaw and Haberman 2003, 2006; Li and Lee 2005), the P-spline approach (Currie et al. 2004), the UN Bayesian approach (Raftery et al. 2013), and the Eurostat EUROPOP2010 forecast (European Commission 2011). Finally, we summarize and discuss our main findings in the last section.

## Methodology of our model

The objective of the proposed model is to forecast mortality for developed populations, even in the presence of changing long-term trends in mortality and dynamic age-shifts of survival improvement. Moreover, the model should be adjustable to different forecast conditions such as a rapid or slow convergence of mortality between a COI and RCs. This flexibility is achieved using optional features (or tools) that can be applied solely or in combination.

### Model input

The proposed model requires death rates by age and calendar time as input. Such detailed mortality data are available for many developed countries in the Human Mortality Database (2013). To gain information about the age pattern of mortality change with time, we calculate the ROMIs for each age-specific death rate over time *ρ*_*x*,*t*_ as in Kannisto et al. (1994):

1 $$ {\rho}_{x,t}=-\left(\frac{m_{x,t+1}}{m_{x,t}} - 1\right) $$

where *m*_*x*,*t*_ denotes the death rate at age *x* in year *t*. Equation 1 is a transformation of the standard equation to estimate the rate of growth (*x*_*t*_ = *x*_0_ *e* ^rt^, see Keyfitz (1977) with *t* = 1). The minus sign ensures that reductions in mortality result in positive values.

**Fig. 1:**
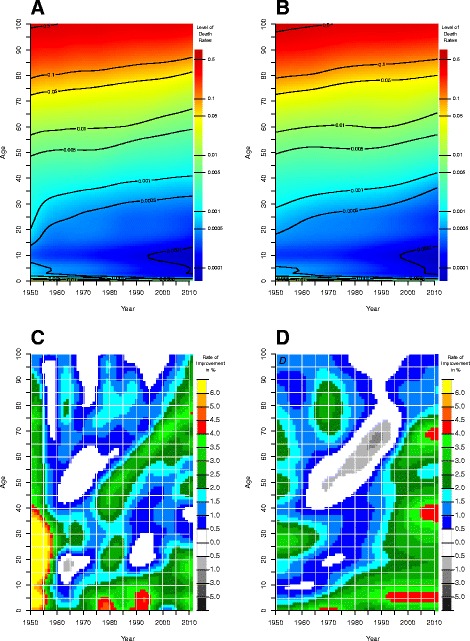
Input of our model. *Upper panel*: (smoothed) death rates for women in the UK (**a**) and Denmark (**b**). *Lower panel*: rates of mortality improvement (in %) for women in the UK (**c**) and Denmark (**d**). Source: Authors’ estimations based on data from the Human Mortality Database (2013)

To illustrate why employing ROMIs might be a better tool than age-specific death rates themselves Fig. 1 depicts in the upper two panels for women in the UK (A) and Denmark (B) death rates on the so-called Lexis surface, i.e., a plane by calendar time and age. We smoothed the death rates with the P-spline smoothing methods of Currie et al. (2004), based on Eilers and Marx (1996) that are implemented in the R package MortalitySmooth of Camarda (2012). Areas with the same color indicate the same level of mortality. The aspect ratio of the two axes has been intentionally chosen so that an increase of 10 years on the calendar time axis matches a 10-year increase on the age axis. Thus, the trajectory of a birth cohort can be followed on a 45° line. Contour lines were added to facilitate orientation on the surface. It is evident in both pictures that a given color tends to reach higher ages over time, corresponding to a decrease in age-specific death rates for women in the UK and Denmark over time. The antagonistic mortality dynamics in both countries, which we could expect from the diverging trajectories for life expectancy at birth, remain somewhat hidden in the two upper panels, though. In our opinion, the display of ROMIs in the two lower panels provides better insights. The various shades of gray indicate a negative improvement; survival worsens in those areas. Minor improvements are shown in blue, and green colors were employed for moderate improvements. Red, orange, and yellow were used for strong declines in mortality. A 3.5% annual decrease in mortality (a green level) translates, for example, to a cut in mortality by half in less than 20 years. The divergent mortality dynamics in the UK and Denmark are clearly more visible in the surfaces of ROMIs (C and D): While mortality decreased rather gradually in the UK in the last decades, Danish women, born approximately *between* the two World Wars, experienced a strong cohort effect with stagnating or even deteriorating survival conditions during the 1980s and early 1990s, which was the primary cause for the decelerated increase in Danish life expectancy at birth in that period (Lindahl-Jacobsen et al. 2016; Jacobsen et al. 2002). In the Appendix, Figure 8 displays the death rates and ROMIs for British and Danish men.

### Core models

We have two optional core models (linear and exponential), which we can use to forecast survival improvements by age and calendar year. Since we use Bayesian inference to run our core models, they automatically model coherence of mortality change among adjacent ages. This ensures that mortality of neighboring ages declines at a similar pace and that mortality gradually increases at adult ages each year. Another advantage is that they capture and quantify forecasting uncertainty, i.e., they give information about the spread and likelihood of our outcome.

#### Core model 1 (linear model)

The first core model is a linear hierarchical model. A hierarchical model can combine within-group and between-group information automatically (Carlin and Louis 2008; Gelman 2006; Gelman et al. 2003; Jackman 2009; Kruschke 2011). In the context of mortality forecasting, this means that such a model can capture the change of mortality within a certain age group as well as between adjacent age groups over time. The level of heterogeneity in mortality across ages determines to what extent they will converge to an overall mean or a similar level. The more mortality differs between adjacent ages over time, the less will the forecasted mortality levels converge to a similar level, and the more will they follow their own trajectory. By contrast, the less mortality differs between adjacent ages over time, the more will the forecasted mortality levels converge to a similar level, and the less will they follow their own trajectory. In the first core model, we forecast ROMIs using a two-level normal model. The trajectory of each ROMI *ρ* for a single age *x* and calendar year *t* follows a linear trend that is specified with age-specific intercepts *β*_1,*x*_ and slopes *β*_2,*x*_:

2 $$ {\rho}_{x,t}\sim N\left({\beta}_{1,x}+{\beta}_{2,x}t,{\sigma}^2\right) $$

The dependency of the ROMIs between adjacent ages *x* is modeled by overall means; *μ*_1_ for the *β*_1,*x*_ and *μ*_2_ for the *β*_2,*x*_:

3 $$ {\beta}_{j,x}\sim N\left({\mu}_j,\tau \right) $$

Combining information about mortality change within a certain age (Eq. 2) and between adjacent ages (Eq. 3) gives a comprehensive picture of mortality improvement across age and time. To capture uncertainty, we assume normally distributed realizations around mean ROMIs using a within age-group variance *σ* ^2^ (Eq. 2) and a between age-group variance *τ* (Eqs. 3 and 4):

4 $$ \tau \leftarrow \mathrm{inverse}\left(\varOmega \right) $$

Moreover, a correlation structure between the intercepts and the slopes is given via the covariance matrix Ω:

5 $$ \varOmega \leftarrow \left(\begin{array}{cc}\hfill {\omega}_1^2\hfill & \hfill \rho {\omega}_1{\omega}_2\hfill \\ {}\hfill \rho {\omega}_1{\omega}_2\hfill & \hfill {\omega}_2^2\hfill \end{array}\right) $$

*ω*_1_ is the standard deviation for the intercepts, *ω*_2_ is the standard deviation for the slopes, and *ρ* is the correlation between the intercepts and the slopes across all ages *x*. We set uniformly distributed priors for the hyper-parameters *σ*, *μ*_1_, *μ*_2_, *ω*_1_, *ω*_2_, and *ρ*:

6 $$ \sigma \sim U\left(0,1\right) $$

7 $$ {\mu}_1\sim U\left(-0.1,0.1\right) $$

8 $$ {\mu}_2\sim U\left(-0.1,0.1\right) $$

9 $$ {\omega}_1\sim U\left(0,1\right) $$

10 $$ {\omega}_2\sim U\left(0,1\right) $$

11 $$ \rho \sim U\left(-1,1\right) $$

These prior specifications are not fixed; they can be adjusted to specific forecast situations. For example, if a forecaster expected only positive survival improvements, she or he could set the uniformly distributed priors for the overall means of the intercepts and slopes to a range of nonnegative values (for more information please see additional notes in the “Model and prior specification” section).

#### Core model 2 (exponential model)

The exponential model can be used (in the medium and long run), when decelerating mortality reductions appear to be more plausible (or more likely to happen) than linearly declining mortality reductions. It models nonlinear trajectories of survival improvements that can also approach long-term minimum or maximum levels. In the second core model, we forecast the trajectory for each age-specific ROMI *ρ*_*x*,*t*_ using an exponential function with log-transformed variables and with normally distributed deviances:

12 $$ {\rho}_{x,t}\sim N\left( \exp \left({\beta}_{1,x}+{\beta}_{2,x} \log (t)\right),{\sigma}^2\right) $$

Normally distributed realizations around the mean *ρ*_*x*,*t*_ can be justified with the fact that the death rates, which are used to compute the *ρ*_*x*,*t*_, usually come from large populations (Schmertmann et al. 2014). The exponential function uses age-specific intercepts *β*_1,*x*_ and slopes *β*_2,*x*_, as well as log-transformed time log(*t*). We set uniformly distributed priors for all standard deviations, i.e., for *σ*, *σ*_1_, and *σ*_2_, that represent uncertainty in the observed mortality data.

13 $$ \sigma \sim U\left(0,1\right) $$

14 $$ {\beta}_{1,x}\sim N\left(\widehat{\theta_{1,x}},{\sigma}_1^2\right) $$

15 $$ {\beta}_{2,x}\sim N\left(\widehat{\theta_{2,x}},{\sigma}_2^2\right) $$

16 $$ {\sigma}_1\sim U\left(0,1\right) $$

17 $$ {\sigma}_2\sim U\left(0,1\right) $$

Moreover, the marginal medians for the *β*_1,*x*_ and *β*_2,*x*_ are estimates for the coefficients *θ*_1,*x*_ and *θ*_2,*x*_ from the linear model

18 $$ \log {\rho}_{x,t}={\theta}_{1,x}+{\theta}_{2,x} \log (t) $$

that we fit to the time series of each age-specific ROMI *ρ*_*x*_ in the base period. To avoid undefined log transformations of negative *ρ*_*x*_-values, we set them to a minimum that is greater than zero. We can also write this linear model as:

19 $$ \widehat{\rho_{x,t}}= \exp \left(\widehat{\theta_{1,x}}+\widehat{\theta_{2,x}} \log (t)\right) $$

to obtain the form that we use in our core model.

#### Implementation

To forecast ROMIs with our core models, we use simulation-based Bayesian inference, i.e., we use the Gibbs sample algorithm (Geman and Geman 1984) to explore the forecasting distribution simulatively. A stationary distribution is reached, if different runs of the Markov chain Monte Carlo (MCMC) simulation converge reliably to similar posterior estimates (Koller and Friedman 2009, p. 509).

To compute and analyze the forecasting distribution, we use the statistical software R (2012) and Just Another Gibbs Sampler (JAGS), a freely available program that can be deployed for Bayesian analysis (Plummer 2012). We use the R-packages rjags (Plummer 2015) and R2jags (Su and Yajima, 2015) to interface between R and JAGS.

JAGS requires certain input data to compute the forecasting distribution of the ROMIs using our core models. Hence, we define our core models in a specific programming language for JAGS. We also provide basic data in R that will be used from JAGS to execute the models. To this basic data belong, for instance, the observed ROMIs, the number of single ages, and the number of forecast years, as well as the age-specific coefficients *θ*_1,*x*_ and *θ*_2,*x*_ for the exponential model. Next to this basic data, we determine several execution properties like the random number generator, the number of parallel chains, the number of iterations, and the number of thinning, as well as the length of the burn-in period. In general, it can be helpful to fit the forecasting models with a large number of chains and iterations to explore the parameter space exhaustively. We use the R-package R2jags to execute parallel chains with JAGS; this can speed up the execution of many iterations. Thinning, by means of saving only each *j*th iteration, can be used to avoid dependencies between adjacent iterations (autocorrelation). To analyze the outcome, we only use the iterations of a chain that has converged to the forecasting distribution. This also means that we use the burn-in period to identify the parameter values of iterations before a chain converges to a constant mean and to exclude them from outcome analysis (King 2012). Running the Gibbs sampling algorithm, as, for instance, described in the “The proposed model” section, we obtain all model parameter estimates simultaneously, and we forecast the mortality trends and their uncertainty coherently. Hence, both core models provide probabilistic mortality forecasts.

### Model output

The output of the proposed mortality forecasting model is simulated posterior distributions for the future ROMIs. These posterior distributions comprise all executed iterations; for instance, when we run the Gibbs sampling algorithm with a total of 5000 iterations, we also obtain a total of 5000 potential future values for each single ROMI. To describe the posterior distribution (and its associated uncertainty), we use quantiles. For example, we use the quantiles *i* of the forecasted ROMIs to forecast the respective quantiles *i* of the death rates *m* by age *x* and time *t*:

20 $$ {m}_{x,t}^i={m}_{x,t-1}^i\left(1-{\rho}_{x,t}^i\right) $$

These forecasted death rates can then be used for further mortality analysis, such as life table calculations. To summarize mortality in a population, we use, for instance, the forecasted death rates to compute life expectancy at birth.

### Additional notes

#### Assuming mortality convergence to capture long-term trend changes

As already motivated in the “Introduction” section, converging mortality between a COI and RCs is a modeling strategy (of the proposed model) that can be optionally applied to forecast long-term trend changes for a COI. In terms of life expectancy at birth, convergence could either mean an accelerated increase for the COI to approach larger values of RCs or a slowdown for the COI to approach smaller values of the RCs. However, the anticipation of trend changes, the selection of RCs, and the combination (or weighting) of multiple population trends are, incontestably, subjective.

Converging mortality implies that a COI and RCs share similar mortality risk factors and survival conditions. Broadly defined groups of countries with similar characteristics may be (highly) developed countries and western countries (Russolillo et al. 2011; Li and Lee 2005; European Commission 2014). Moreover, the Epidemiological Transition by Omran (1971) and the Health Transition by Frenk et al. (1991) may serve as a theoretical basis to justify the assumption of mortality convergence between a COI and RCs. If a COI and RCs belong to the same group and the COI simply lags some years behind and is expected to catch up in coming years, we recommend to use mortality trends of vanguard RCs (of the same group) in order to close the gap and to forecast converging mortality between the COI and RCs. This also works in the opposite direction: If the COI is some years ahead and it is expected to slowdown in coming years, we recommend to use mortality trends of lagging RCs (of the same group) in order to forecast converging mortality.

Mortality trends of the COI and RCs can be combined outside of the core models in order to determine different pathways of mortality convergence; a simple way to do this is to gradually increase the weight of the RCs to one and to gradually decrease the weight of the COI to zero in the forecast years. More sophisticated weighting schemes could be designed, including time delays for variable onsets of converging mortality in the years to forecast.

#### Model and prior specification

Since the model specifications in the “Core models” section aim to be convenient to forecast mortality for many (developed) populations, we use weakly informative prior distributions to “regularize” the posterior distribution and to keep it roughly within reasonable bounds (Gelman et al. 2014, p. 52). Although we are aware that choosing weakly informative priors is fraught with difficulties, the experiences we have made with uniform distributions have been quite positive. Moreover, prior sensitivity analyses in the “Diagnostics” section indicate that the results (or forecasts) of our model are only marginally sensitive to various prior specifications, including other distributions, such as the Beta, Gamma, and Normal distribution. However, users of our model could adjust the priors so that they better reflect the given mortality conditions and their expectations for a population of interest.

#### Potential limitations and future enhancements

The modeling of dynamic mortality changes via survival improvements and mortality convergence via RCs makes our model particularly suitable to forecast mortality (1) extrapolatively, if mortality in a COI is expected to continue regularly, or (2) extrapolatively and explanatorily, if mortality in a COI is expected to change and to approach the mortality of RCs. This strength also exposes the potential weakness of our model, since it is less suitable to forecast mortality for a country, if a past trend is expected to change in a way that has not yet been observed in any population. This might be the case in, for instance, East Germany and Russia, two countries which experienced an unprecedented fall/rise in mortality after the dissolution of the Soviet Union. To overcome this limitation, we could use expected or target mortality change schedules, a strategy similar to (optimal) model life tables, which have been suggested by the United Nations (1982) for developing countries. Another possible enhancement would be a data-driven choice of (1) RCs and (2) combinations of multiple mortality trends. The model could find a compromise between trends of the COI and RCs via, for example, time series cointegration. Similarly to King and Soneji (2011), we could also add a covariate representing mortality improvement in a RC with a lag of *n* years (instead of a risk factor such as obesity or smoking 25 years back in time). Despite the fact that such model specifications would reduce the subjective character of our proposed model, they would probably impede the modeling of expected long-term trend changes in forecast years if past data do not already incorporate them. This is mainly because such specifications would identify similar (or common) trends rather than expected trend changes. Future analyses might reveal the pros and cons of such alternative model specifications.

## Application of our model

In this section, we apply the proposed model to forecast mortality of British and Danish women and men, and we compare its predictive ability with those of other, well-established models. We are aware of the fact that such comparisons are insufficient to fully assess the accuracy of the applied approaches (Hyndman et al. 2013); however, they are useful to give a first impression how each approach deals with regular mortality developments in the UK and irregular mortality trends in Denmark.

An obvious choice for comparative purposes is the model of Lee and Carter (1992) due to its world-wide acceptance and application in the field of mortality forecasting (Booth et al. 2006; Shang et al. 2011). Numerous enhancements of this model have been proposed since its introduction about 20 years ago. For our comparison, we select three generalizations suggested by Renshaw and Haberman (2003; 2006) who add terms regarding cohort and extra-period effects. We also employ the coherent variant of Li and Lee (2005). Apart from the class of those Lee-Carter models, we also compare our model with the Bayesian approach of Raftery et al. (2013) that is used by the UN Population Division for the World Population Prospects 2012 (United Nations 2013) and with the one proposed by Currie et al. (2004) that is based on a two-dimensional, nonparametric smoothing using P-splines (Eilers and Marx 1996).

All models are applied and tested using the different mortality trends in the UK and Denmark. British women and men feature a rather regular mortality development with a stable increase in life expectancy at birth in the last decades. Danish women, by contrast, experienced a period of virtual stagnation during the 1980s until the middle of the 1990s, caused by the widespread prevalence of smoking of women born between the two World Wars (Lindahl-Jacobsen et al. 2016; Jacobsen et al. 2002; Christensen et al. 2010). In addition, the slow increase in life expectancy at birth for Danish men since the 1960s has strengthened in the early 1990s. The detection of such structural changes in mortality trends has been analyzed recently (Vallin and Meslé 2009; van Berkum et al. 2013) and poses additional obstacles to forecast mortality accurately.

We generate validating and prospective mortality forecasts for British and Danish women and men with all models. Since the base period has a large effect on mortality forecasts (Stoeldraijer et al. 2013; Janssen and Kunst 2007), we use at least as many reference years as we have years to forecast. Moreover, we take the same base year in the validating and prospective forecasts in order to include similar past trends. The validating forecasts from 1991 to 2011 are based on data from 1965 to 1990. They can be used to assess the predictive ability of each model by simply comparing the estimates of the models with the observed values. We expect that the models perform similarly well in the British case due to the continuation of the past trend in the forecast years. By contrast, we expect greater performance differences between the models in the Danish case due to the long-term trend change in the forecast years. This accelerated increase in Danish female and male life expectancy at birth might be better captured by the coherent approaches (i.e., the model of Li and Lee (2005) and the proposed model) that can include faster mortality reductions of other populations (that still belong to the same group such as highly developed countries). The prospective forecasts from 2012 to 2050 are based on death rates between 1965 and 2011. As we do not know how mortality will develop in the future, we cannot compare forecasted and observed mortality data. Alternatively, we compare the mortality forecasts of our model with those of other approaches and agencies like Eurostat.

Comparing the performance of such different methods, which use extrapolation, explanation, and/or expectation to forecast mortality, can be fair as long as concrete assumptions like the base period and the length of the forecast horizon are equal (Stoeldraijer et al. 2013). Since the concrete assumptions are equal in the British and Danish mortality forecasts, the approaches only differ in their method and in their method-related assumptions, which can be, for instance, the transformation of death rates regarding age groups (of single or 5 years of age), smoothing, differentiating, the usage of fitted or observed jump-off rates, or the assumption of converging mortality. Since all these approaches forecast (total) mortality, use the same death rates from the Human Mortality Database (2013) for the same base period and forecast horizon, we reckon that differences in the forecasts are only due to these method-related differences. In the past, such comparisons have been conducted only for extrapolative methods (Booth et al. 2006; Shang et al. 2011) but also for extrapolative and explanatory methods (Stoeldraijer et al. 2013), including methods that even use etiological data such as smoking and nonsmoking-related mortality (Janssen et al. 2013; Wang and Preston 2009).

### Data and parameter settings

#### The proposed model

To forecast mortality with the proposed model for women and men in the UK and Denmark, we take death counts and exposure times for single ages (0 to 100+) and years from the Human Mortality Database (2013). Following Vallin and Meslé (2004), we assume that mortality of Danish women and men will increasingly converge to the lower overall mortality of other highly developed countries. In the validating forecast, we take mortality data from Sweden, and in the prospective forecast, we also include data from France, Italy, and Japan. We compute ROMIs for each age-specific death rate over time; the death rates are smoothed using P-splines (Currie et al. 2004; Eilers and Marx 1996). We then forecast the survival improvements with our second core model via JAGS by letting the Gibbs sample algorithm run with five parallel chains for a total of 5200 iterations after a burn-in period of 200 iterations. The run-length and related settings rely on the Raftery-Lewis diagnostic and trace plots, which are presented in the “Diagnostics” section. To avoid dependence between adjacent trials, we only save each fifth iterate for inferences. In addition, we exclude implausibly low and high forecasts for ROMIs by setting thresholds, i.e., they can neither fall below 0.005 nor can they exceed 0.035. These thresholds rely on observed values (see Fig. 1), and they imply a mortality decline for all ages at a minimum of 0.5% and at a maximum of 3.5%. An annual mortality improvement of 3.5% is extraordinarily high and means that mortality can be reduced by half in less than 20 years. In case the model approaches minimum or maximum ROMIs, the forecasted death rates still decline with time. Appendix Table 3 summarizes all parameter settings for our mortality forecasts.

#### Model of Lee and Carter (LC)

The mortality forecasts of the LC model are estimated with the R-package *demography* of Hyndman et al. (2015), which requires death rates and exposures by age and time, arranged in a *demogdata* object, as input. In the *lca* function, we set the parameter *adjust* to *dt* to adjust the time index *k*_*t*_ to the total number of deaths when the model is refitted. We then use the *forecast* function to forecast mortality with the estimated model and to compute prediction intervals.

#### Models of Renshaw and Haberman (RH)

We generate mortality forecasts with three extensions of the LC model proposed by Renshaw and Haberman (2003; 2006), applying the R-packages *ilc* (Butt and Haberman 2010b) and *demography* (Hyndman et al. 2015). We fit the three models, labeled as *h0*, *h1*, and *h2*, with the *lca.rh* routine that needs death rates and exposures, arranged in a *demogdata* object, as input. For most parameters, we use the default settings. Exceptions are parameters determining the type of the model and the error structure; we set them to *h0*, *h1*, or *h2*, and to *Gaussian* or *Poisson*. In addition, we set the *spars*-parameter to the recommended value of 0.6, which is intended to smooth the input data. To forecast the fitted models, we use the *forecast*-function, setting the *jump.choice*-parameter to *actual*, which produces more plausible mortality forecasts than setting it to *fit*. The output contains forecasted death rates and life expectancies at birth. In our comparative analysis, we only take the parameter settings for each of these three models, which achieve the best forecasting results.

#### P-spline approach of Currie et al. (P-spline)

The P-spline approach of Currie et al. (2004) is used to smooth and forecast mortality over age and time; it models death counts with the natural logarithm of the exposed population as an offset in a Poisson setting. We employ the software package *MortalitySmooth* by Camarda (2012). Two parameters are of crucial importance: the smoothing parameter(s) *λ* and the order of the penalty. The *λ*s are found by optimizing the BIC on a large grid of potential *λ* values. *Specifying the correct order of the penalty is less straightforward, as pointed out in the paper* (Currie et al. 2004, p. 289, 297). An order of the penalty of 1 refers to future mortality at a constant level, 2 to improvements at a constant rate, and 3 to an accelerating rate. After experimentation, we choose the default order of the penalty of 2, which gives the best results in our validating framework.

#### Coherent model of Li and Lee (LC-coherent)

To estimate the coherent model of Li and Lee (2005), we use the web-based platform *LCFIT* that is hosted on the website of Berkeley’s demography department. The interface requires mortality rates and population counts for at least two countries. We combine the Danish data with death rates and exposures from Sweden in the validating forecast, and, additionally, with mortality data from France, Italy, and Japan in the prospective forecast. Moreover, experimentation has shown that the default settings of the optional parameters produce the best results.

#### Bayesian approach of Raftery et al. (BayesLife)

We use the R-package *bayesLife* (Ševčíková and Raftery 2015) to forecast mortality with the Bayesian approach of Raftery et al. (2013). Applying the *run.e0.mcmc* and the *e0.predict* routine with default parameter values, we simulate 160,000 iterations using three chains. The exploration of the trajectories of future life expectancy at birth yields quantiles for quinquennial data, i.e., in the validating forecasts, this approach provides life expectancy at birth for the 5-year periods 1990–1995, 1995–2000, 2000–2005, and 2005–2010 of which we take the mid-points 1993, 1998, 2003, and 2008.

### Validating forecast

#### Prediction intervals

**Fig. 2:**
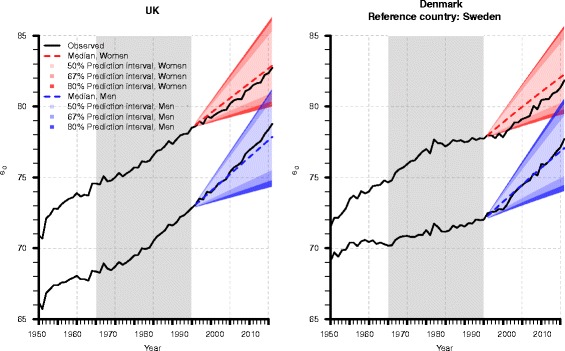
Observed (*black*) and forecasted life expectancy (*e*_0_) of our model for British (*left*) and Danish (*right*) women (*red*) and men (*blue*); increasing forecast uncertainty is represented by the widening 80, 67, and 50% prediction intervals, whose colors become brighter from the outside to the median (*dashed line*). In this validating forecast, we take the data from 1965 to 1990 (*gray colored box*) as basis to forecast mortality from 1991 to 2011. Moreover, in the Danish forecasts, we combine the mortality trends of Denmark and Sweden

Figure 2 displays the probabilistic mortality forecasts of our model from 1991 to 2011; it describes them with the median and the 50, 67 and 80% prediction intervals for life expectancy at birth of women and men in the UK and Denmark. Our model forecasts increasing life expectancy at birth for both sexes; the observed values fluctuate (narrowly) around the median estimates. Forecast uncertainty increases with time, too; this effect is indicated by gradually widening prediction intervals. For example, with a probability of 80%, British female life expectancy at birth will be between 79 and 82 years in 2000 and between 80 and 86 years in 2011. Thus, the width of the 80% prediction interval becomes twice as large between the tenth and the twenty-first forecast year, i.e., it increases from 3 to 6 years. Figure 13 (in the Appendix) demonstrates that the prediction intervals of the proposed model are comparable to those of the other approaches. In 2011, the width of the 95% prediction intervals ranges between 4.6 and 6.5 years among the models.

The calibration of prediction intervals can also be assessed using empirical frequencies (Gneiting et al. 2007; Raftery et al. 2013). Tables 1 and 2 list the percentage of observed female and male life expectancy values at age zero, respectively, that is captured in the 95 and 80% prediction intervals from 1991 to 2011. For example, in the forecast for Danish women, the 95 and 80% prediction intervals capture 91 and 81% of the observed values according to our model, and 100 and 86% of the observed values according to the LC model. An exception is the P-spline approach that has some difficulties to catch the changing mortality trend for Danish women. As a consequence, only 62% of the observed values fall in the 95% prediction interval of this approach. Please note that empirical frequencies are not given for the LC-coherent model in the British case. That is because we apply this model only when we combine mortality trends of multiple populations, i.e., when we assume converging mortality between Denmark and the RC. One hundred and 95% of the observed values of Danish female life expectancy at birth fall in the 95 and 80% prediction interval of the LC-coherent forecast, respectively; the empirical frequencies are smaller for Danish men: only 29 and 24% fall in the 95 and 80% prediction interval. The h1 model of RH achieves better results than the h0 and h2 variants; its empirical frequencies are larger for the UK than for Denmark and for women than for men. For example, 95 and 47% of the observed values for British and Danish women fall in the 80% prediction interval, respectively. A 100% coverage of observed values indicates that some of the 95 and 80% prediction intervals are too wide, but it could also indicate that the actual development does not get lost due to too narrow prediction intervals. The assessment of such findings is also related to conceptual and practical issues; when building (robust) models, a forecaster is often torn between generality, realism, and precision (Levins 1966; Orzack 2012). Most models are either general, realistic, or precise. A 100% coverage of observed values of 95 and 80% prediction intervals could imply robust models, which are on the one side harder to differentiate, but on the other side more reliable.

**Table 1:** Empirical frequencies to assess calibration of prediction intervals in the validating mortality forecasts for British and Danish women

Prediction interval:	United Kingdom		Denmark	
	95%	80%	95%	80%
Our model	100	100	91	81
LC-Hynd	100	100	100	86
P-spline	100	91	62	33
LC-coherent	–	–	100	95
RH-h0	5	0	0	0
RH-h1	100	95	100	47
RH-h2	100	24	5	0

**Table 2:** Empirical frequencies to assess calibration of prediction intervals in the validating mortality forecasts for British and Danish men

Prediction interval:	United Kingdom		Denmark	
	95%	80%	95%	80%
Our model	100	100	95	95
LC-Hynd	76	62	14	0
P-spline	100	100	71	67
LC-coherent	–	–	29	24
RH-h0	20	0	0	0
RH-h1	81	29	48	5
RH-h2	0	0	0	0

#### Median forecasts

**Fig. 3:**
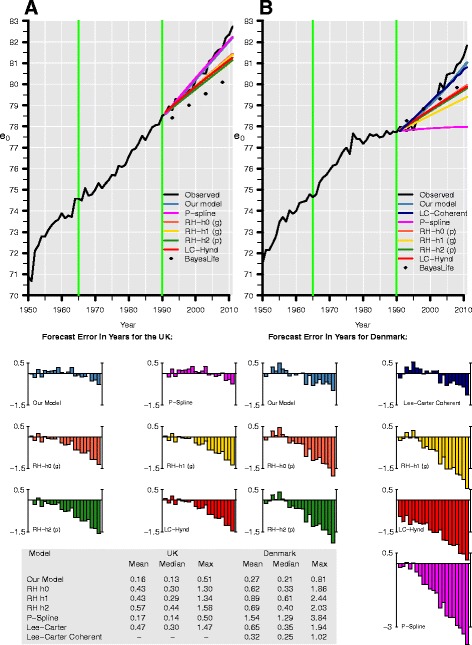
Observed (*black line*) and forecasted life expectancy at birth (*e*_0_) of our model (*blue*), the P-spline approach (*magenta*), the LC model (*red*), four of its variants—h0 (*light red*), h1 (*yellow*), h2 (*green*), and coherent (*navy blue*—and of the UN Bayesian approach (*squares*) for women in the UK (*left*) and Denmark (*right*). The validating forecasts from 1991 to 2011 rely on data from 1965 to 1990 (*green vertical lines*). We combine the mortality trends of Danish and Swedish women in our model as well as in the coherent LC forecast. In the RH models, (p) and (g) denote Poisson and Gaussian errors, respectively. Forecast errors are shown for all forecasts

Figure 3 depicts the median forecasts for British and Danish female life expectancy at birth for all models. The green vertical lines indicate the beginning (1965) and end (1990) of the base period that was used for the validating forecasts from 1991 to 2011. We compare the predictive ability of all approaches with forecast errors; they are defined as the difference between forecasted and observed life expectancies at birth *e*_0_:

21 $$ {E}_t={e}_{0,t}^{\mathrm{forecast}}-{e}_{0,t}^{\mathrm{observed}} $$

In Fig. 3, the forecast errors are displayed for each model below the two panels. In the case of the UK (left panel), the P-spline model and our model provide a satisfactory fit; their errors fluctuate around zero, resulting in mean absolute errors of 0.17 and 0.16 years, respectively. Considering that (record) life expectancy at birth increases annually at a pace of about 0.25 years, this deviation is negligibly small. By contrast, the errors of the LC model and its extensions of RH are somewhat greater and they accumulate with time. Their mean absolute error ranges between 0.43 and 0.57 years, and they underestimate life expectancy at birth in 2011, the end of our validating forecasting period, by 1.30 to 1.58 years.

As pointed out previously, Denmark’s life expectancy at birth (right panel) is challenging to forecast due to a trend change in the forecast years. Surprisingly, the nonparametric P-spline approach produced the largest forecast errors. Neither modifying the order of the penalty nor adding weights, which change over time, improves the P-spline forecast. The LC model and its variants of RH perform better; the approaches assuming converging mortality capture the trend change in the forecast years even better. That is why the maximum absolute errors of the coherent forecast of Li and Lee (2005), 1.02 years, and of the proposed model, 0.81 years, are so low compared to the other approaches. The results are also given for British and Danish men in Figure 9 in the Appendix; the pattern of the errors are similar to the results for women; however, they are more pronounced for men. In addition, Figure 10 in the Appendix depicts the coherent forecasts for Danish women and men assuming a convergence to mortality of Sweden, France, Italy, and Japan (as in the prospective forecasts in the next section). These forecasts show that both the coherent LC model and the proposed model are sensitive to the choice of RCs. That the proposed model is sensitive to the choice of RCs is also shown, for example, in a mortality forecast for the USA (Bohk and Rau, 2016). Moreover, the forecasts of remaining life expectancy at age 65 are given for the proposed model in Figure 11 in the Appendix.

### Prospective forecast

#### Prediction intervals

**Fig. 4:**
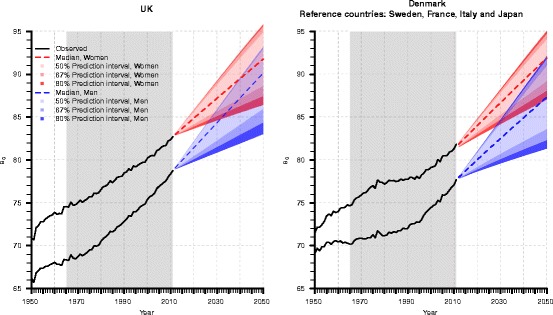
Observed (*black*) and forecasted life expectancy at birth (*e*_0_) of our model for British (*left*) and Danish (*right*) women (*red*) and men (*blue*); increasing forecast uncertainty is represented by the widening 80, 67, and 50% prediction intervals, whose colors become brighter from the outside to the median (*dashed line*). We forecast mortality from 2012 to 2050 given data from 1965 to 2011 (*gray colored box*). Moreover, in the Danish forecasts, we combine the mortality trends of Denmark, France, Italy, and Japan

Figure 4 displays the probabilistic mortality forecasts of the proposed model from 2012 to 2050; increasing magnitude of life expectancy at birth and its associated uncertainty are illustrated using the median and gradually widening 80, 67, and 50% prediction intervals for women and men in the UK and Denmark. In 2050, female life expectancy at birth ranges between 86.39 and 95.81 years in the UK and between 87.02 and 95.06 years in Denmark with a probability of 80%. It is remarkable that the difference between the lower quantiles is larger than the difference between the upper quantiles, indicating that the uncertainty (or the variability) is greater for slower increases than for stronger increases in life expectancy at birth in these two countries. The same pattern can be observed in the male forecasts.

#### Median forecasts

**Fig. 5:**
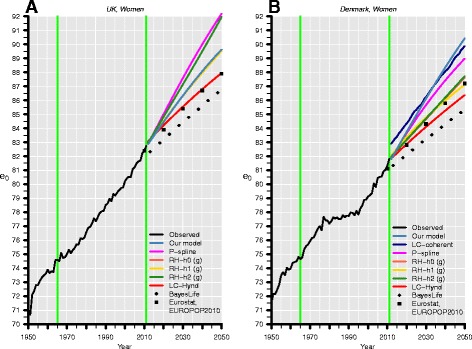
Observed (*black line*) and forecasted life expectancy at birth (*e*_0_) of our model (*blue*), the P-spline approach (*magenta*), the LC model (*red*), four of its variants—h0 (*light red*), h1 (*yellow*), h2 (*green*), and coherent (*navy blue*)—the UN Bayesian approach (*triangles*), and of Eurostat (*squares*) for women in the UK (*left*) and Denmark (*right*). The forecasts from 2012 to 2050 rely on data from 1965 to 2011 (*green vertical lines*). We combine mortality trends of Danish, Swedish, French, Italian, and Japanese women in our model as well as in the coherent LC model. In the RH models, (p) and (g) denote Poisson and Gaussian errors, respectively

Figure 5 displays the median forecasts of British and Danish female life expectancy at birth from 2012 to 2050 of the proposed model; the LC model; its extensions h0, h1, and h2; its coherent variant; the P-spline approach; the UN Bayesian approach; and the Eurostat EUROPOP2010 forecast (European Commission 2011). For each model fit, we take the death rates from 1965 to 2011 as basis (green vertical lines).

Comparing the prospective mortality forecasts reveals several aspects. First, all models predict that the increase in life expectancy at birth is likely to continue. Models applying the LC predictor structure forecast stronger improvements for British than for Danish women. For example, they predict that British women will gain on average seven additional years of life until 2050, while Danish women are forecasted to gain only five additional years of life. This is probably due to the weak survival improvements during the 1980s and early 1990s in Denmark. Thus, the trend in the base period has a decisive influence on a forecast, particularly for purely data-driven methods that extrapolate only past trends. The LC-coherent model and the proposed model forecast a stronger growth in Danish life expectancy at birth than the other models; that is because they assume that Danish mortality will converge to the lower mortality trends of Swedish, French, Italian, and Japanese women. Additional years of life until 2050 are forecasted to be 8.6 years according to our model and 7 years according to the LC-coherent model. Although both approaches combine the same mortality trends in their forecasts, the transition between observed and forecasted values appears to be more plausible (or smooth) for our model than for the coherent model; at least we were unable to remove this jump-off bias using the web-based platform LCFIT. Second, it is striking that our model is not restricted to predict only moderate gains or only large gains in life expectancy at birth. In the British case, our model forecasts moderate gains, and in the Danish case, it forecasts strong increases in life expectancy at birth compared to the other approaches. By contrast, the UN Bayesian approach and the LC model provide rather conservative forecasts for British and Danish women. Third, the forecasted values for life expectancy at birth vary considerably between the models. For example, the P-spline approach forecasts one of the greatest values for life expectancy at birth in 2050: For British women, it is 92.18 years, and for Danish women, it is 88.97 years. By contrast, the LC model forecasts life expectancy at birth to be relatively low in 2050: For British women, it is 87.95 years, and for Danish women, it is 86.36 years. Only the UN Bayesian approach forecasts even smaller values for life expectancy at birth: For British women, it is 86.54 years, and for Danish women, it is 85.12 years (in 2048). Fourth, each model provides prediction intervals to capture and quantify forecast uncertainty. For example, the 95% prediction interval of our model and of the P-spline approach is relatively wide: In 2050, the life expectancy at birth of British women is estimated to range between 85 and 96 years according to our model and between 87 and 96 years according to the P-spline approach. By contrast, the other models provide smaller prediction intervals. For example, the LC model predicts British female life expectancy at birth to range between 85 and 90 years with a probability of 95%. The prospective median forecasts are also given for men in Figure 12 in the Appendix; the forecasted trends are similar to those described for women.

**Fig. 6:**
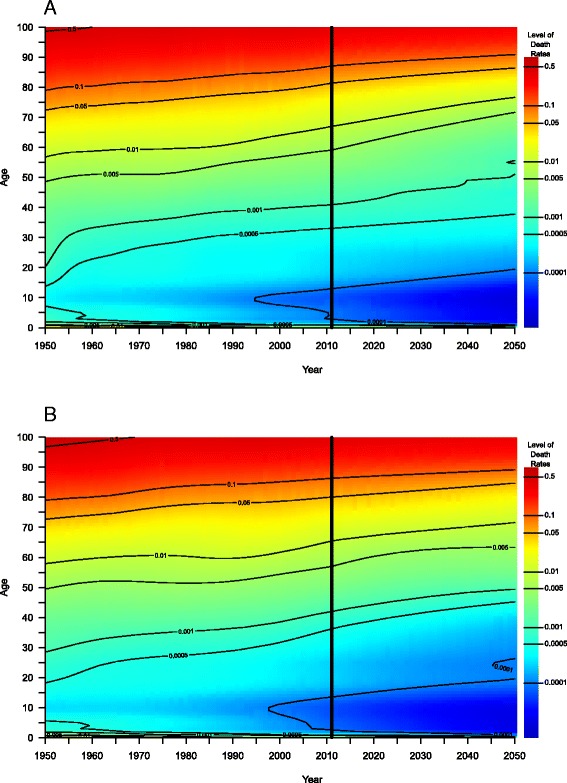
Death rates by single age; observed in the calendar years 1950 to 2011 and forecasted with the exponential core model in the years 2012 to 2050 for women in the UK (*top*) and Denmark (*bottom*). The transition from observed to forecasted death rates is illustrated by the *vertical black line* in both displays

Figure 6 depicts the death rates of the proposed model for British (top) and Danish (bottom) women between 1950 and 2050. A closer look at these two panels, based on smoothed input data, reveals at least two things. First, there is a smooth transition between the observed and forecasted death rates around the year 2011 (vertical line), i.e., there is no jump-off bias. Second, the death rates decline gradually for each age over time; this is indicated by the fine color gradient for each age and the slightly increasing contour lines. Contour lines represent how a certain level of mortality proceeds over age with time; since they show no erratic fluctuations, they indicate that low mortality levels successively reach older ages. The plots also illustrate that the forecasts of our model are biologically plausible and that they preserve the natural age schedule of mortality. For example, adult mortality increases with age, i.e., mortality at age 71 does not drop below the level at age 70 in a given year. Figure 14 (in the Appendix) depicts the predicted death rates of our linear core model. The results are similar to those from the exponential model, displayed in Fig. 6. In the last section, we briefly discuss the benefits of both core models.

### Diagnostics

In this section, we assess if the Gibbs sampler converged to the correct posterior distribution (via trace plots and autocorrelation functions), if the run-length is sufficient (via the Raftery-Lewis diagnostic (Raftery and Lewis 1992)), as well as if the forecasts are (in) sensitive to the prior specifications.

**Fig. 7:**
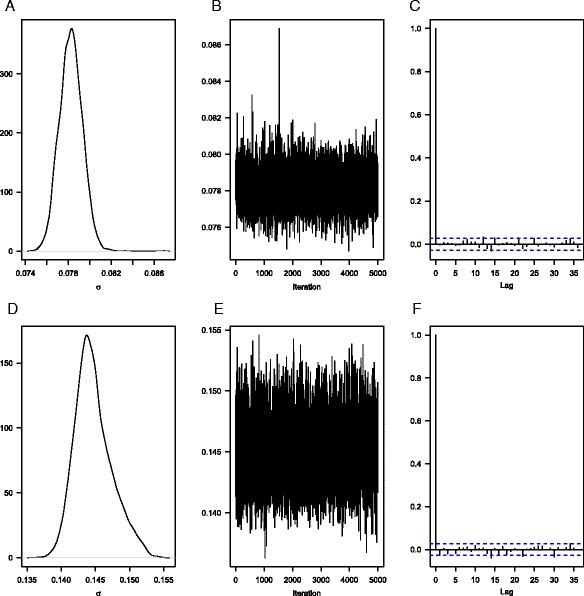
Marginal posterior density function (**a**, **d**), traceplot (**b**, **e**), and autocorrelation function (**c**, **f**) for the parameter σ in the British (*top*: **a**–**c**) and Danish (*bottom*: **d**–**f**) validating forecast of our model after 5000 iterations

**Fig. 8:**
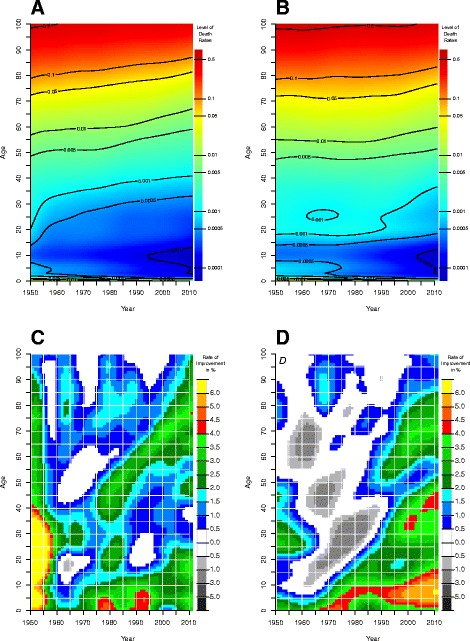
Input of our model. *Upper panel*: death rates (smoothed) for men in the UK (**a**) and Denmark (**b**). *Lower panel*: rates of mortality improvement (in %) for men in the UK (**c**) and Denmark (**d**). Source: Authors’ estimations based on data from the Human Mortality Database (2013)

**Fig. 9:**
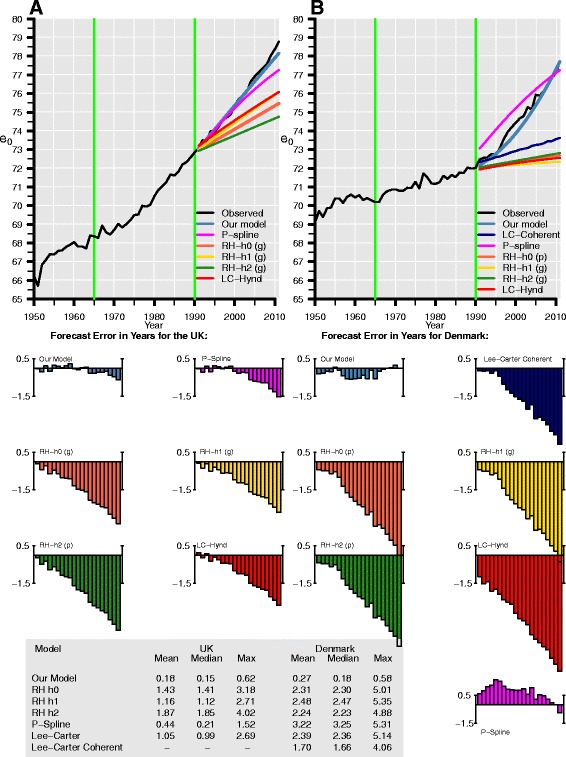
Observed (*black line*) and forecasted life expectancy at birth (*e*_0_) of our model (*blue*), the P-spline approach (*magenta*), the LC model (*red*), and four of its variants, h0 (*light red*), h1 (*yellow*), h2 (*green*), and coherent (*navy blue*), for men in the UK (*left*) and Denmark (*right*). The validating forecasts from 1991 to 2011 rely on data from 1965 to 1990 (*green vertical lines*). We combine the mortality trends of Danish and Swedish men in our model as well as in the coherent LC forecast. In the RH models, (p) and (g) denote Poisson and Gaussian errors, respectively. Forecast errors are shown for all forecasts

**Fig. 10:**
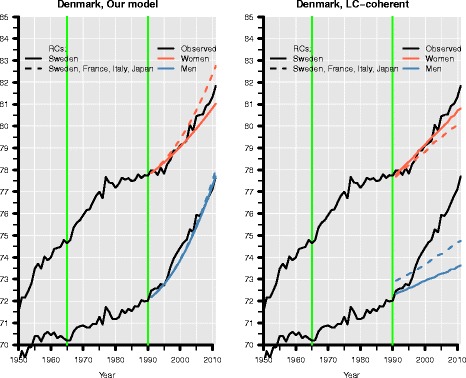
Sensitivity of forecasted life expectancy at birth of the LC-coherent model (*right*) and the proposed model (*left*) for Danish women (*red*) and men (*blue*) using the RC Sweden (*solid lines*) and, in addition, France, Italy, and Japan (*dashed lines*). Observed data are depicted in *black*

**Fig. 11:**
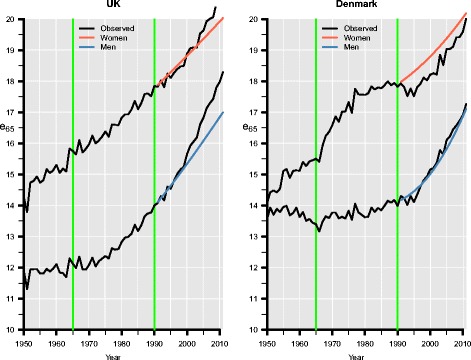
Validating forecasts of remaining life expectancy at age 65 for British (*left*) and Danish (*right*) women (*red*) and men (*blue*) of the proposed model. Observed data are depicted in *black*

**Fig. 12:**
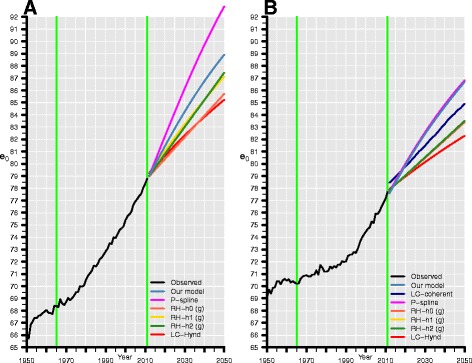
Prospective forecasts of life expectancy at birth for British (*left*) and Danish (*right*) men of the proposed model (*blue*), the P-spline approach (*magenta*), the LC model (*red*), and four of its variants: h0 (*light red*), h1 (*yellow*), h2 (*green*), and coherent (*navy blue*). Observed data are depicted in *black*. In the Danish forecast, the proposed model and the coherent LC model use data from Sweden, France, Italy, and Japan

**Fig. 13:**
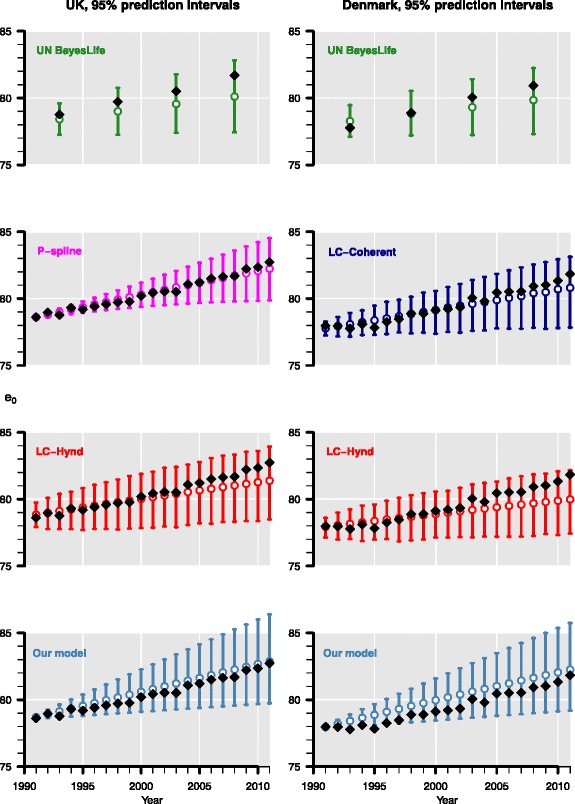
Validating forecasts from 1991 to 2011, given data from 1965 to 1990, of our model (*blue*), the LC model (*red*), its coherent variant (*dark blue*), the P-spline method (*magenta*), and of the UN Bayesian approach (*green*) for British (*left*) and Danish (*right*) women. Observed life expectancy (*e*_0_) is represented by *black squares*, the median forecasts by *white circles*, and the 95% prediction intervals by the *vertical lines*. We combine the mortality trends of Danish and Swedish women in our model as well as in the coherent LC model

**Fig. 14:**
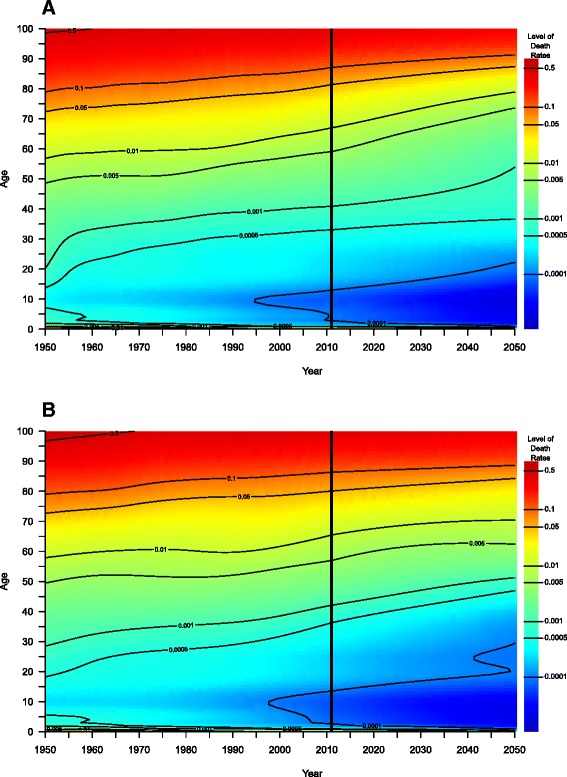
Death rates by single age; observed in the calendar years 1950 to 2011 and forecasted with our linear core model in the years 2012 to 2050 for women in the UK (*top*) and Denmark (*bottom*). The transition from observed to forecasted death rates is illustrated by the *vertical black line* in both displays

Figure 7 exemplarily depicts the marginal posterior density function and the traceplot, as well as the autocorrelation function for the single model parameter *σ*, the within age-group variance, of the validating forecasts for British (upper panels: a–c) and Danish women (lower panels: d–f). The marginal density functions and the traceplots indicate that *σ* converges to a stationary level, i.e., the parameter values oscillate around a constant value so that we can assume that the parameter space has been explored exhaustively. The autocorrelation functions indicate that *σ* is relatively good mixing because they decrease exponentially and stay at a low level for higher lags (King 2012).

Appendix Table 4 lists the results of the Raftery-Lewis diagnostic, i.e., the number of iterations needed for each model parameter to obtain an accurate estimate for the 0.025 quantile with a probability of 0.95. For the UK as well as for Denmark, the estimated run-length *N* fluctuates between 3561 and 3741 for all model parameters, and the dependence factor *I* fluctuates between 0.951 and 0.999. As neither the run-length *N* nor the dependence factor *I* has exceptionally high or low values for certain model parameters, we conclude that 5000 iterations will be sufficient to estimate an accurate projection outcome with our model.

A simple prior sensitivity analysis suggests that our British and Danish mortality forecasts are relatively insensitive to the (rather weakly informative) prior specifications. To demonstrate this, we repeatedly generate validating forecasts with our model using different prior specifications, i.e., we use the Uniform, the Beta, and the Normal distribution with different parameter values to determine various levels for their location and spread. Appendix Figures 15 and 16 show that these different prior specifications result in (very) similar marginal posterior densities and, beyond that, Appendix Figure 17 shows that these prior specifications also yield (very) similar forecasts for British (left) and Danish (right) female life expectancy at birth from 1991 to 2011. These findings suggest that the results of our model are mainly data-driven.

## Discussion

Advances in mortality forecasts will become even more important in the future than they are already today. In many highly developed countries, improving chances of survival are the main driver of population aging (Preston and Stokes 2012); the increase in life expectancy at birth is due to mortality reductions at all ages, though the highest gains in mortality reductions advance to successively older ages (Christensen et al. 2009). Some forecasts underestimate this ongoing progress in mortality reductions from younger to older ages (Ševčíková et al. 2016; Li et al. 2013). In the past, this failure would have meant to underestimate mortality of people at working ages; in the future, it would probably lead to an underestimation of mortality of people aged 65 and older (because survival improvements will increasingly reach those ages). This will have an increasing impact on social welfare systems. Underestimating mortality of people at working ages corresponds to having more people who pay premiums. Underestimating mortality of people in retirement ages, however, will result in having more people who receive benefits for longer periods than previously anticipated.

Some approaches, like the seminal model of Lee and Carter and several of its extensions, extrapolate past mortality trends with a time-invariant age schedule of mortality change and may induce, therefore, systematic forecast errors (e.g., Booth et al. 2006). These forecast errors can become even larger if a country experiences long-term trend changes in the forecast years. We illustrate this effect in validating mortality forecasts for women and men of the UK and Denmark from 1991 to 2011, given data from 1965 to 1990. While life expectancy at birth increased for British females and males at a regular pace, it almost stagnated for Danish women in the 1980s and early 1990s, and increased thereafter at an accelerated pace. Danish men also experienced a trend change in life expectancy at birth in the 1990s; the slow increase of the previous decades started to accelerate thereafter. Since we forecast mortality for only two countries, we cannot generalize the differences in the performance of the applied models. However, they provide a useful first indication how each approach deals with (ir)regular mortality developments in a medium forecast horizon of roughly 20 years. Our results suggest that models assuming time-invariant age profiles of survival improvement appear to systematically underestimate life expectancy at birth for British women and men, and even stronger for Danish women and men. The proposed model addresses these issues using a novel combination of two modern concepts in mortality forecasting in a Bayesian framework:

1. Age-specific ROMIs can capture dynamic age-shifts of mortality change over time. ROMIs were employed in recent studies by Mitchell et al. (2013) and Haberman and Renshaw (2012), who used them to extend the predictor structure of the LC model.
2. Converging mortality between a COI and RCs. This can be useful for populations, which undergo irregular mortality developments and that face long-term trend changes in the forecast years. RCs who belong to the same group as the COI, e.g., western industrialized countries, or that will be in the same state of Health Transition as the COI (Frenk et al. 1991; Vallin and Meslé 2004) can be a good choice. Recent works by, for example, Li and Lee (2005), Cairns et al. (2011), and Russolillo et al. (2011), show that it can be worthwhile to jointly forecast mortality for two or more populations, especially when they exhibit similar characteristics concerning health and mortality.

Applying these tools, the validating forecasts of the proposed model mirror the observed life expectancies at birth of women and men in the UK and Denmark with only small deviations. In the case of the UK, the proposed model benefits from using ROMIs, which allow a time-variant age pattern of mortality change. Only the P-spline approach, proposed by Currie et al. (2004), forecasts British mortality with deviations so small that they are similar to those of the proposed model. In the case of Denmark, the proposed model benefits not only from ROMIs but also from convergence between Danish and Swedish mortality trends. According to Vallin and Meslé (2004), we expect that Denmark will approach the lower overall mortality of Sweden in the forecast years. The coherent model of Li and Lee (2005) forecasts Danish mortality with comparably small deviations as the proposed model; that is because we also combined the Danish with the Swedish trend in this forecast.

We interpret these findings as a first indication that the proposed model can generate valid forecasts for developed countries experiencing (ir)regular mortality trajectories; a thorough validation will be part/object of follow-up analyses possibly including mortality forecasts for more countries using different base periods, forecast horizons, core models, and RCs. Apart from comparing probable future lifespans among countries, such analyses will primarily expose strengths and weaknesses of this methodology (under certain forecast conditions) as well as user guidelines. However, relying on the presented results here, we can give initial recommendations regarding some selection criteria in order to generate mortality forecasts (for developed countries) with the proposed model. First, our model appears to be applicable for forecast horizons up to a length of 50 years. Second, two points appear to be especially relevant for the selection of a core model. The first point is that, contrary to the linear model, the exponential model cannot forecast mortality increases, since the logarithm of negative ROMIs is not defined. The second point is that the linear model extrapolates temporal trends for survival improvements at each age ad infinitum, whereas the exponential model can also forecast a decelerating mortality decline. This may be important for currently steep increases in survival improvements as well as for longer forecast horizons. Third, the subjective choice of RCs is certainly worth discussing. Although purely data-driven methods are objective, they typically fail to forecast trend changes in coming years if they have not been observed in past data; they rather extrapolate the mean trend of the base period. (This can be seen in the validating mortality forecasts for Denmark of the non-coherent LC models in the “Validating forecast” section). To circumvent this problem, the proposed model can assume that mortality of a COI will converge to the mortality of RCs. Regarding the selection of appropriate RCs, we recommend to choose countries that belong to the same broad group as the COI; such a group can be, for example, western industrialized countries. Moreover, the theory of Health Transition by Frenk et al. (1991) or the Epidemiological Transition by Omran (1971) can be used along with major risk factors affecting morbidity and mortality, such as the smoking epidemic (Lopez et al. 1994; Thun et al. 2012; Stoeldraijer et al. 2014; Janssen et al. 2015), to justify or explain the choice of RCs for a COI. Fifth, the proposed model also allows the estimation of purely hypothetical settings, such as how would mortality in country XY develop if it had the (past) mortality experience of country YZ. Such a conditional projection could reveal, for example, how mortality would develop in a COI if the health care conditions of another country would have applied.

In summary, we propose a framework, which appears to be applicable to derive mortality forecasts for developed populations experiencing stable and unstable mortality developments. Since it is uncertain what will happen in the future, the proposed model provides tools that can be switched on or off to adjust to certain forecast conditions. To these optional features belong, for example, the core models (linear or exponential) and the assumption of mortality convergence. This optional (plug-in) setting also simplifies further extensions of the proposed model in terms of the integration of additional functions. Despite promising initial forecasts for the UK and Denmark, as well as for Italy, Spain, West Germany (Bohk and Rau 2014), and the USA (Bohk and Rau 2016), our model will be further validated in a next step.

## Figures and Tables

**Fig. 15:**
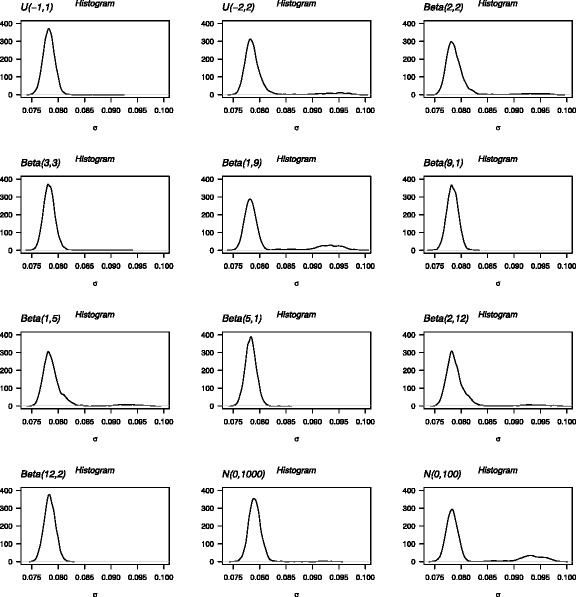
Prior sensitivity analysis: Marginal posterior densities for *σ* in the validating forecasts for British women of our model using different prior specifications. We use the Uniform, the Beta, and the Normal distribution with different parameter values to determine various levels of their location and spread. Despite these different prior specifications, the resulting marginal posterior densities are (very) similar

**Fig. 16:**
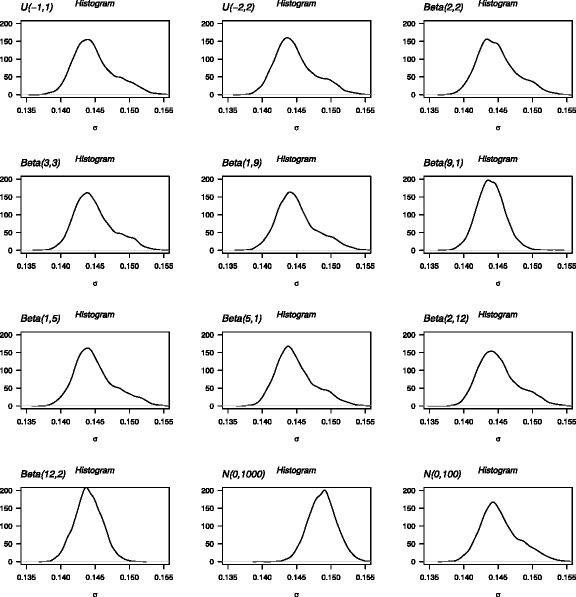
Prior sensitivity analysis: marginal posterior densities for *σ* in the validating forecasts for Danish women of our model using different prior specifications. We use the Uniform, the Beta, and the Normal distribution with different parameter values to determine various levels of their location and spread. Despite these different prior specifications, the resulting marginal posterior densities are (very) similar

**Fig. 17:**
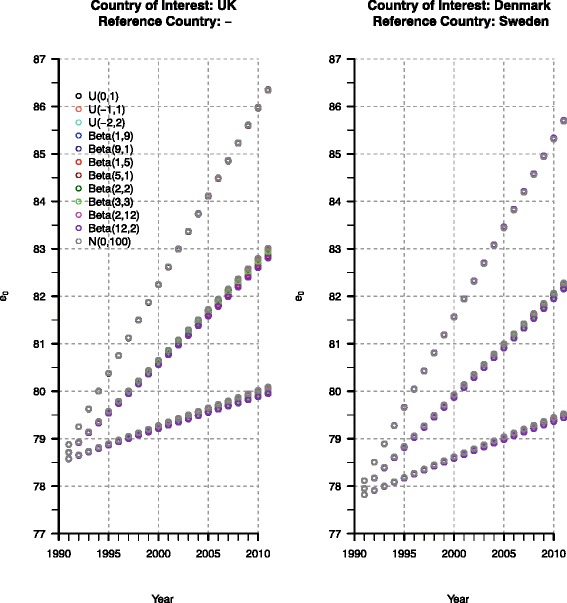
Prior sensitivity analysis: the validating forecasts (represented by the median and the 80% prediction intervals) of British (*left*) and Danish (*right*) life expectancy are (very) similar, although we use different prior specifications (differently colored *circles*) for our model

## Appendix

**Table 3:** Model parameters and their values in the mortality forecasts for British and Danish women and men

Model parameter	Parameter values
Country of interest	UK/Denmark
Reference country (validating)	UK/Sweden
Reference country (prospective)	UK/Sweden, France, Italy, Japan
Minimum age	0
Maximum age	110+
Base period (validating)	1965–1990
Base period (prospective)	1965–2011
Forecast horizon (validating)	21 years (1991–2011)
Forecast horizon (prospective)	39 years (2012–2050)
Core model	Exponential
Number of iterations	5200
Number of adaptions	200
Number of parallel chains	5
Number of thinning	5
Adjust forecasted *ρ*_*x*,*t*_	TRUE; (min: 0.005; max: 0.035)

**Table 4:** Raftery-Lewis diagnostic for single parameters of our validating mortality forecasts for women in the UK and Denmark

		*σ*	*σ*_1_	*σ*_2_
United Kingdom	*N*	3741	3741	3561
	*I*	0.999	0.999	0.951
Denmark	*N*	3680	3680	3680
	*I*	0.982	0.982	0.982

## Abbreviations

COI: Country of interest
JAGS: Just another Gibbs sampler
LC: Lee-Carter
MCMC: Markov chain Monte Carlo
RC: Reference country
RH: Renshaw-Haberman
ROMI: Rate of mortality improvement
